# Cartilage thickness distribution and its dependence on demographic, radiographic, and MRI structural pathology in knee osteoarthritis—data from the IMI-APPROACH cohort

**DOI:** 10.1007/s00256-025-04907-4

**Published:** 2025-03-21

**Authors:** Mylène P. Jansen, Tom D. Turmezei, Kishan Dattani, Dimitri A. Kessler, Simon C. Mastbergen, Margreet Kloppenburg, Francisco J. Blanco, Ida K. Haugen, Francis Berenbaum, Wolfgang Wirth, Felix Eckstein, Frank W. Roemer, James W. MacKay

**Affiliations:** 1 Department of Rheumatology & Clinical Immunology, University Medical Center Utrecht, Utrecht, The Netherlands; 2 Department of Radiology, Norfolk , Norwich University Hospital, Norwich, UK; 3 Norwich Medical School, University of East Anglia, Norwich, UK; 4 Department of Radiology, University of Cambridge, Cambridge, UK; 5 Artificial Intelligence in Medicine Lab (BCN-AIM), Facultat de Matemátiques I Informática, Universitat de Barcelona, Barcelona, Spain; 6 Department of Rheumatology, Leiden University Medical Center, Leiden, The Netherlands; 7 Clinical Epidemiology, Leiden University Medical Center, Leiden, The Netherlands; 8 Grupo de Investigación de Reumatología (GIR), Centro de Investigación CICA, Departamento de Fisioterapia y Medicina, INIBIC – Complejo Hospitalario Universitario de A Coruña, SERGAS, Universidad de A Coruña, A Coruña, Coruña, Spain; 9 Center for Treatment of Rheumatic and Musculoskeletal Diseases (REMEDY), Diakonhjemmet Hospital, Oslo, Norway; 10 Department of Rheumatology, AP-HP Saint-Antoine Hospital, Paris, France; 11 Sorbonne University, INSERM CRSA, Paris, France; 12 Center for Anatomy and Cell Biology & Ludwig Boltzmann Institute for Arthritis and Rehabilitation (LBIAR), Research Program for Musculoskeletal Imaging, Paracelsus Medical University, Salzburg, Austria; 13 Chondrometrics GmbH, Freilassing, Germany; 14 Quantitative Imaging Center, Department of Radiology, Boston University School of Medicine, Boston, MA USA; 15 Department of Radiology, Universitätsklinikum Erlangen and Friedrich-Alexander-Universität Erlangen-Nürnberg (FAU), Erlangen, Germany

**Keywords:** 3D, Cartilage thickness, Osteoarthritis, Pathology, Knee

## Abstract

**Objective:**

Cartilage surface mapping is a technique that can visualize 3D cartilage thickness variation throughout a joint without a need for arbitrary regional definitions. The objective of this cross-sectional study was to utilize this technique to evaluate the cartilage thickness distribution in knee osteoarthritis patients and to analyze to what extent it depends on demographic, radiographic, and MRI structural pathology strata.

**Methods:**

Patients of the IMI-APPROACH cohort were included, with MRIs obtained at 1.5 T or 3 T. Tibial and femoral cartilage segmentation and registration with a canonical surface were performed semi-automatically. Kellgren-Lawrence and OARSI grading were performed on knee radiographs; MOAKS scoring was performed on MRI scans. The association of demographics and radiographic and MRI scorings with cartilage thickness distribution was analyzed with general linear models using statistical parametric mapping.

**Results:**

Two hundred eighty-seven patients were included. Male sex and height were positively associated with cartilage thickness particularly in the trochlea and medial femur, respectively, with differences up to 0.5 mm (male vs female), while radiographic joint space narrowing and bone marrow lesions showed region-specific negative associations (up to 0.14–0.5 mm per grade). Kellgren-Lawrence grade, MOAKS meniscal extrusion, and osteophytes showed patterns of positive and negative associations, with increasing grades showing reduced local tibiofemoral cartilage thickness, but greater thickness in the trochlea (both up to 0.2–0.3 mm per grade).

**Conclusions:**

Decreased height, female sex, and increasing tibiofemoral pathology were associated with thinner tibiofemoral cartilage. Unexpected results such as consistently thicker cartilage in the anterior femur with increasing disease or osteophytosis states provide opportunities for future research.

**Supplementary Information:**

The online version contains supplementary material available at 10.1007/s00256-025-04907-4.

## Introduction

Knee osteoarthritis (OA) is characterized by cartilage loss, along with other structural pathology such as meniscal extrusion and formation of osteophytes or bone marrow lesions (BMLs) [1, 2]. How the cartilage thickness varies in relation to demographic factors, such as sex, or other OA pathology, such as BMLs or meniscal extrusion, has been a topic of interest in several previous studies [3–6]. Research thus far has focused mainly on regional or subregional analyses in especially the medial and lateral tibiofemoral compartment, evaluating cross-sectional or longitudinal variations in knee OA cohorts such as the Osteoarthritis Initiative (OAI) or Innovative Medicine’s Initiative Applied Public–Private Research enabling OsteoArthritis Clinical Headway (IMI-APPROACH) [7–13]. Unconstrained by subregional boundaries, cartilage surface mapping (CaSM) is a quantitative 3D analytic method that can demonstrate visually how cartilage thickness varies across a joint [14]. It also allows visualization of the influence of other parameters on cartilage thickness variations throughout the joint. Therefore, it can be especially useful in indicating new areas of interest within a joint and generating new hypotheses and paths of research to pursue. As such, the purpose of this cross-sectional study was to explore the relationship between 3D cartilage thickness distributions in knee OA and demographic, radiographic, and MRI structural pathology factors. Such data would provide further support for the construct validity of this approach to cartilage analysis as well as generate hypotheses for future research.

## Methods

### Patients

Patients from the prospective observational IMI-APPROACH cohort were used [15]. The cohort contains extensive data of 297 people with clinical knee OA according to American College of Rheumatology (ACR) criteria, including from five European centers. While data was collected over 2 years of follow-up, only baseline data was used for the current study. The index knee of all patients was determined by which knee met ACR criteria; if both knees met the criteria, the most affected knee as indicated by the patient based on the severity of complaints was chosen as the index; when both knees were affected similarly, the right knee was chosen.

### Imaging

Weight-bearing posteroanterior knee radiographs of the index knee were acquired according to the Buckland-Wright protocol [16]. From the radiographs, Kellgren-Lawrence (KL) grading (0–3) and Osteoarthritis Research Society International (OARSI) scoring of the medial (0–3) and lateral (0–3) joint space narrowing (JSN) were performed by one experienced observer [17, 18].

Additionally, a 1.5 T or 3 T MRI scan of the index knee was acquired, including a 3D SPGR sequence for quantitative cartilage thickness analysis and axial, sagittal, and coronal intermediate weighted fat suppressed sequences for semi-quantitative MRI Osteoarthritis Knee Scores (MOAKS) [12, 13]. MOAKS scoring was performed by one experienced radiologist (FWR; > 10 years of experience with MOAKS scoring) and included scoring (0–3) of the following: meniscal extrusion (medial and lateral anterior and exterior); BMLs in the patellofemoral (PF; four subregions), medial tibiofemoral (medial TF; five subregions) and lateral TF (five subregions) compartments; osteophytes in the PF (six subregions), medial TF (three subregions), and lateral TF (three subregions) compartments. MOAKS scoring and descriptive results of structural involvement of knees in IMI-APPROACH have been described in more detail previously; MOAKS intra-observer variability analysis of the used parameters in this cohort showed almost perfect agreement (all weighted Kappa ≥ 0.84) [13, 19].

### Cartilage surface mapping

The validation and initial clinical application of CaSM as an analysis technique have been evaluated previously [14]. In the current study, analyses were performed using the 3D SPGR scans. First, a deep learning model was used to automatically generate a pre-segmentation of the femoral and tibial bone and outer whole-joint cartilage contours [20]. All contours were checked and, where necessary, manually adjusted using Stradview (University of Cambridge Department of Engineering, Cambridge, UK, in-house developed software freely available at https://mi.eng.cam.ac.uk/Main/StradView), by two observers (MPJ, KD) trained by the same experienced radiologist (JWM). Next, the inner and outer cartilage surfaces were detected automatically in Stradview via model-based deconvolution of the image intensity data along a normal to the outer cartilage surface. The cartilage thickness was measured at each vertex of the surface by calculating the distance between the outer and inner cartilage surfaces. All cartilage of the femur and tibia was included (i.e., no regional boundaries were imposed). This was done for every scan for patches of the femur, medial, and lateral tibia separately; the process has previously been described in more detail [14, 21].

The obtained (outer) surfaces and corresponding thickness values were registered to canonical (template) surfaces using an initial similarity transformation and subsequent thin-plate spline registration in wxRegSurf (University of Cambridge Department of Engineering, Cambridge, UK, in-house developed software freely available at http://mi.eng.cam.ac.uk/∼ahg/wxRegSurf/) to allow for comparison between participants and aggregate data from the whole study cohort.

### Statistical analyses

MATLAB R2024a and the SurfStat MATLAB package (https://www.math.mcgill.ca/keith/surfstat/, modified for compatibility with Stradview-generated surfaces by Graham Treece of the University of Cambridge) were used for vertex-wise analysis and visualization of the whole-joint cartilage thickness. The dependence of cartilage thickness on demographics, radiographic, and MRI structural pathology strata was evaluated with statistical parametric mapping (SPM; part of SurfStat). This method can use thickness values at each vertex in general linear models and deliver *p*-values corrected for multiple comparisons, using the minimum of threshold of random field theory and false discovery rate corrections. The output of SPM is a visualization of regions of the canonical surface where the influence of the factor of interest is statistically significant (*p* < 0.05) according to vertex-wise significance testing. The dependence on demographics (sex, age, BMI, height, and weight) was first analyzed individually (i.e., in separate models) and then in multivariable models for simultaneous adjustment for other demographics (i.e., including all demographics as independent variables in the same model; BMI was not included due to its strong correlation with height and weight to prevent multicollinearity issues). The dependence on different radiologic (KL grade, medial JSN, lateral JSN) and MRI pathology strata (medial meniscal extrusion, lateral meniscal extrusion, medial BMLs, lateral BMLs, PF BMLs, medial osteophytes, lateral osteophytes, PF osteophytes) was analyzed separately for each regional pathology and was all corrected for relevant demographics. Since JSN is known to be significantly affected by meniscal extrusion, additional analyses were performed where the association between JSN and cartilage thickness was adjusted for ipsilateral meniscal extrusion. For pathologies where scoring was performed in multiple subregions (MOAKS), the maximum score present in each compartment was taken as the compartmental score. For additional subregional analyses of MOAKS scores, the association of cartilage thickness with the presence/absence of a pathology (binary) was analyzed instead of the original score, to avoid results being overly influenced by a small number of high scores. Subregional analyses were not performed for pathologies that were present in too few patients (*n* < 20) to avoid reliance on a limited group.

## Results

### Cohort overview

In total, 287 patients had baseline MRI scans and could be analyzed. An overview of participants’ demographics and structural tissue damage on a compartmental level is shown in Table 1. For the presence of subregional pathology, see Supplementary Table S1.

**Table 1:** Demographic and pathology overview

Parameter	Total (*n* = 287)
Demographics	
Male sex, *n* (%)	64 (22)
Age, mean ± SD	66.4 (7.1)
BMI, mean ± SD	28.0 (5.2)
Height, mean ± SD	167.1 (10.0)
Weight, mean ± SD	78.2 (15.8)
Radiographic pathology, *n* (%)	
KL grade	
- 0	54 (19)
- 1	76 (27)
- 2	63 (22)
- 3	84 (29)
- 4	10 (4)
JSN lateral > 0	43 (15)
JSN medial > 0	132 (47)
MRI pathology > 0, *n* (%)	
Meniscal extrusion, medial	168 (60)
Meniscal extrusion, lateral	44 (16)
BMLs, medial	99 (35)
BMLs, lateral	68 (24)
BMLs, patellofemoral	162 (56)
Osteophytes, medial	225 (79)
Osteophytes, lateral	182 (64)
Osteophytes, patellofemoral	190 (67)

*SD* standard deviation, *BMI* body mass index, *KL* Kellgren-Lawrence, *JSN* joint space narrowing, *BML* bone marrow lesion

**Fig. 1:**
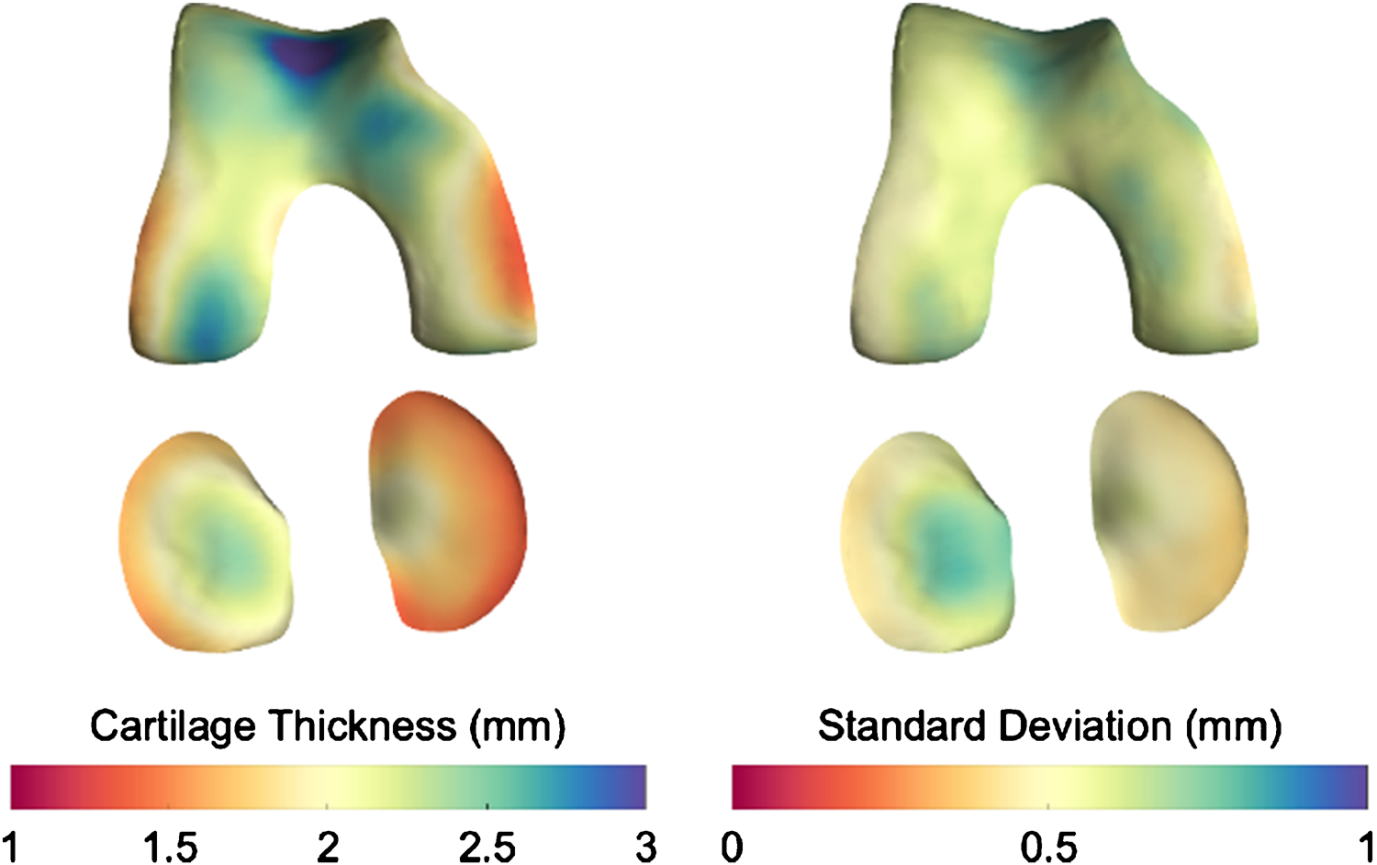
Average cartilage thickness (left) and standard deviation (right)

On average across the cohort, patients had thinner cartilage on the medial side of the tibiofemoral articular surfaces, with the exterior region having a minimum thickness of approximately 1.5 mm on average across participants. In contrast, the cartilage was thicker on the central lateral side of the joint and in the trochlea (Fig. 1).

### Dependence on demographics

In univariable models, male patients showed significantly thicker cartilage throughout the entire joint with a difference of up to 0.5 mm compared to female patients (Supplementary Figure S1). Age was negatively associated with cartilage thickness particularly in the trochlea (anterior femur) and tibia, with 10 years older in age corresponding with up to 0.35-mm thinner cartilage. While BMI was not significantly associated with cartilage thickness, weight and especially height showed a significant positive association throughout large parts of the joint; 10 cm greater height indicated up to 0.25-mm thicker cartilage across nearly all surfaces.

**Fig. 2:**
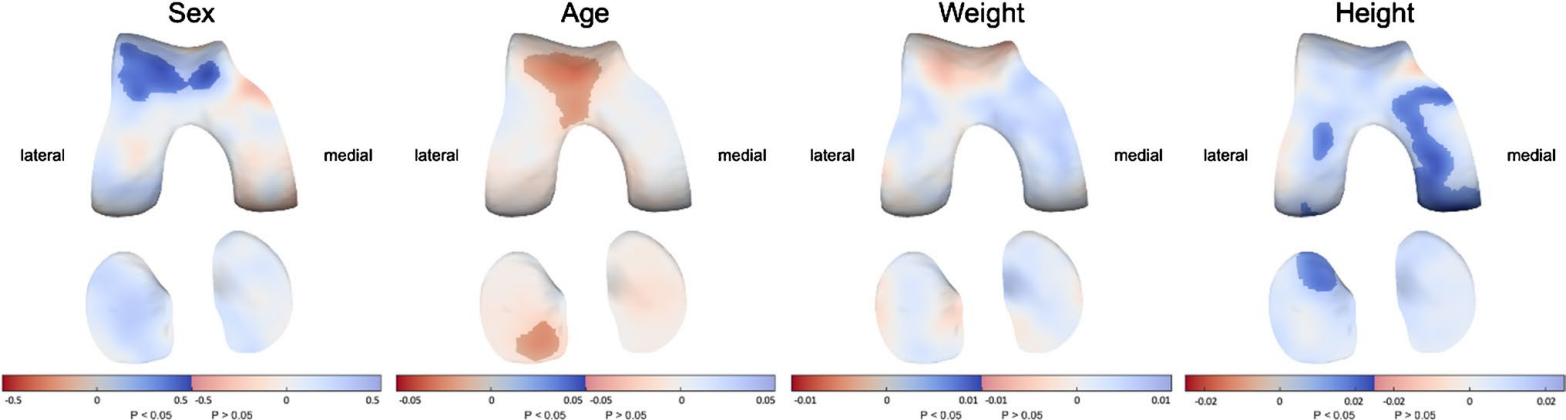
The association of demographics with cartilage thickness distribution in multivariable models. Statistically significant differences (*p* < 0.05) are indicated by the unmasked areas, while washed-out areas indicate non-significant differences (*p* > 0.05). Blue indicates an increase and red a decrease in cartilage thickness as a result of male sex (vs female sex, showing up to 0.5-mm thicker cartilage in men), higher age (per year increase, showing up to 0.35-mm thinner cartilage for a 10-year increase in age), higher weight (per kg increase, showing no significant association), higher height (per cm increase, showing 0.25-mm thicker cartilage for a 10 cm increase in height). Note: color scale differs between models

Evaluating sex, age, weight, and height in multivariable models (where all four parameters are included as independent variables; Fig. 2), weight was no longer significantly associated with cartilage thickness. Furthermore, the positive association of height with cartilage thickness seemed limited to the lateral and particularly medial tibiofemoral compartment, but not the patellofemoral compartment, while male patients only showed significantly thicker cartilage in the trochlea.

Based on these results, all pathology models were adjusted for sex, age, and height.

### Radiographic pathology

**Fig. 3:**
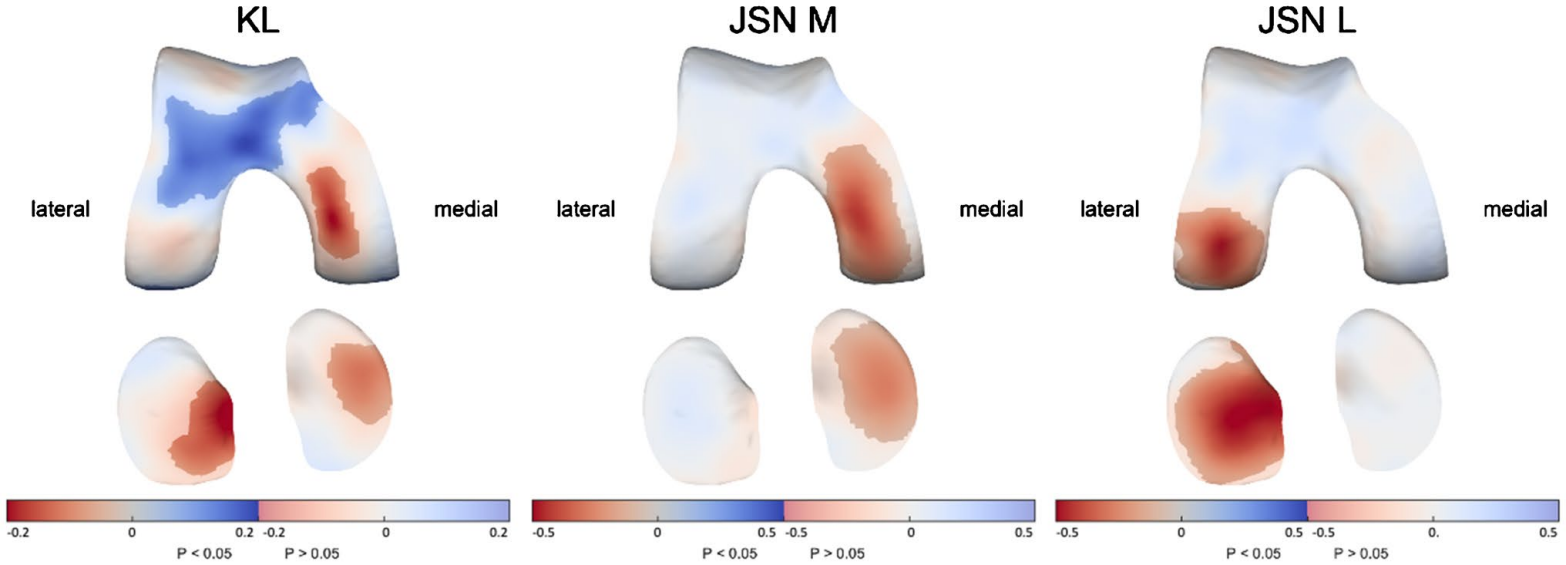
The association of radiographic pathology with cartilage thickness distribution. Statistically significant differences (*p* < 0.05) are indicated by the unmasked areas, while washed-out areas indicate non-significant differences (*p* > 0.05). Blue indicates an increase and red a decrease in cartilage thickness as a result of higher Kellgren-Lawrence (KL, up to 0.2 mm change in both directions per KL grade increase) or medial (M, up to 0.4-mm thinner cartilage per JSN grade increase) or lateral (L, up to 0.5-mm thinner cartilage per JSN grade increase) joint space narrowing (JSN) grades. Note: color scale differs between models

A higher KL grade was associated with significantly thinner cartilage in the medial femur and medial and lateral tibia as well as thicker cartilage in the anterior femur (up to 0.2 mm per KL grade in both directions; Fig. 3). Medial JSN was associated with significantly thinner cartilage in the medial compartment (up to 0.4 mm per JSN grade), while lateral JSN showed similar effects in the lateral compartment (up to 0.5 mm per JSN grade). Additionally correcting the JSN models for ipsilateral meniscal extrusion showed that the significant effects largely remain the same, indicating that JSN and cartilage thickness are significantly negatively associated even when taking meniscal extrusion into account.

### Meniscal extrusion

**Fig. 4:**
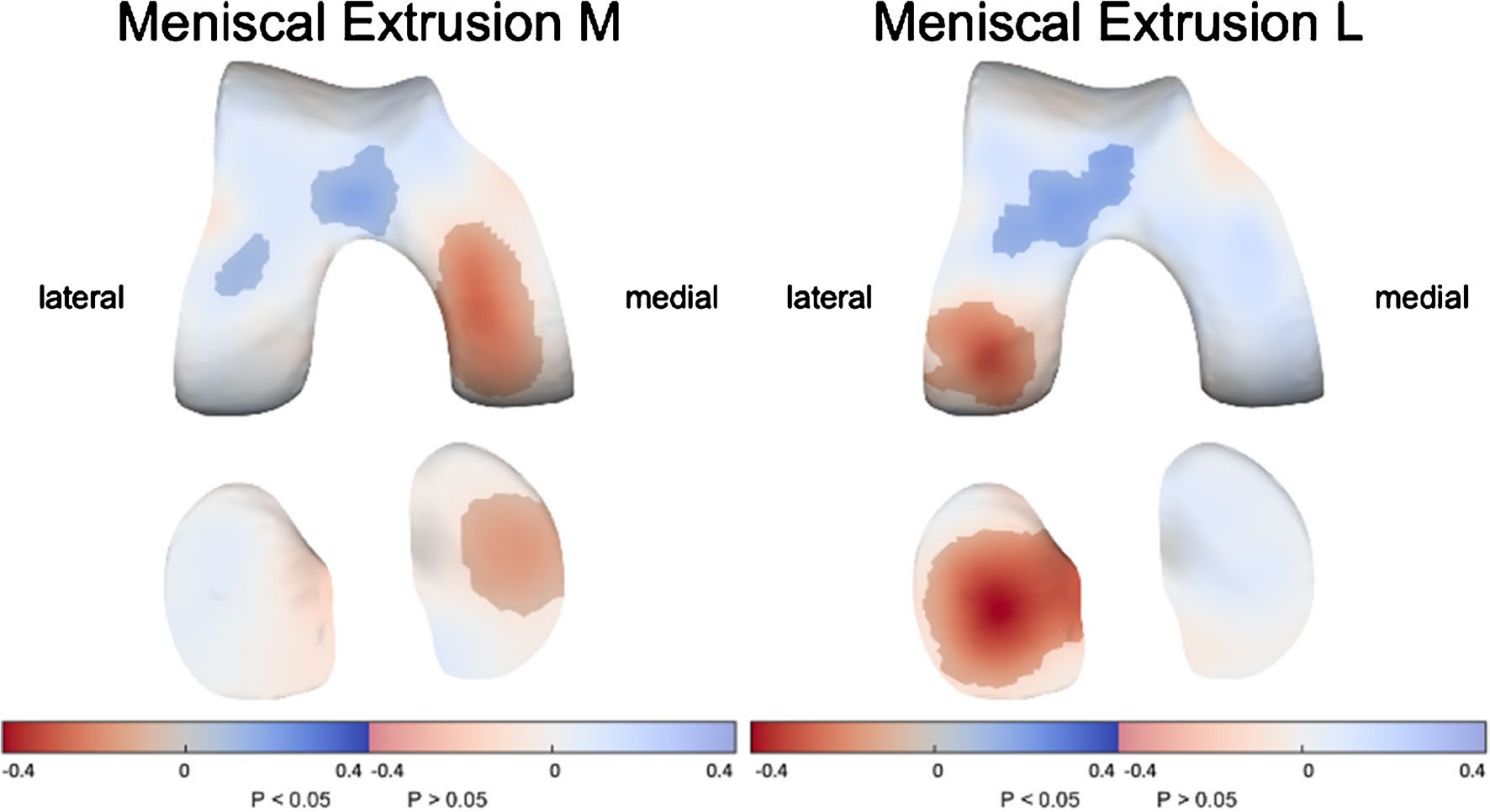
The association of meniscal extrusion with cartilage thickness distribution. Statistically significant differences (*p* < 0.05) are indicated by the unmasked areas, while washed-out areas indicate non-significant differences (*p* > 0.05). Blue indicates an increase and red a decrease in cartilage thickness as a result of a higher medial (M, up to 0.3-mm thinner cartilage per grade increase) or lateral (L, up to 0.4-mm thinner cartilage per grade increase) extrusion grade

Medial meniscal extrusion was associated with thinner cartilage in the medial femur and tibia (up to 0.3 mm per grade) while lateral meniscal extrusion was associated with thinner cartilage in the lateral femur and tibia (up to 0.4 mm per grade overall; Fig. 4). For both sides, patients with more extensive meniscal extrusion showed somewhat thicker cartilage in the anterior femur.

Evaluating the two subregions where medial meniscal extrusion is scored separately (anterior and medial) based on the presence/absence of extrusion (i.e., score 0 vs > 0) showed that both directions of meniscal extrusion showed similar effects (Supplementary Figure S2). This analysis could not be performed for lateral meniscal extrusion due to the small number of patients with lateral anterior meniscal extrusion.

### Bone marrow lesions

**Fig. 5:**
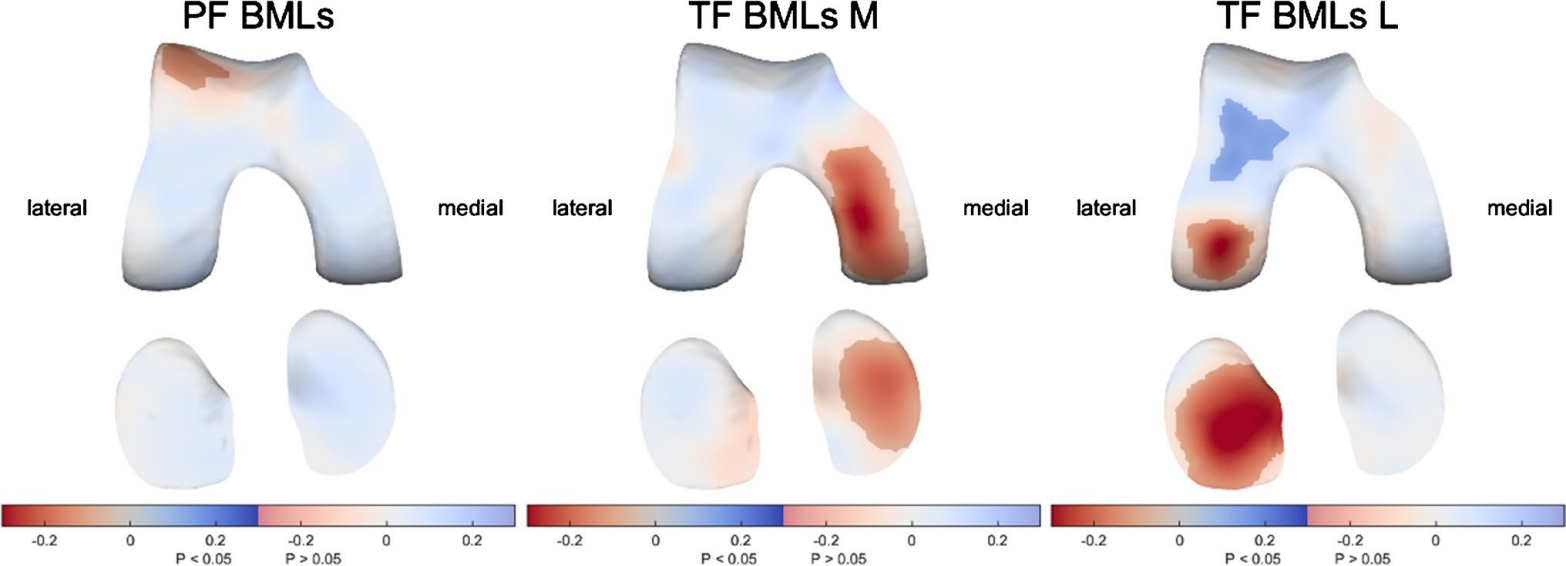
The association of bone marrow lesions (BMLs) with cartilage thickness distribution. Statistically significant differences (*p* < 0.05) are indicated by the unmasked areas, while washed-out areas indicate non-significant differences (*p* > 0.05). Blue indicates an increase and red a decrease in cartilage thickness as a result of a higher patellofemoral (PF, up to 0.14-mm thinner cartilage per grade increase), medial (M, up to 0.32-mm thinner cartilage per grade increase) tibiofemoral (TF), or lateral (L, up to 0.35-mm thinner cartilage per grade increase) TF BML grade

BMLs were associated with significantly thinner cartilage in corresponding regions, although for the PF region, this seems limited to the lateral side (up to 0.14 mm per grade) and weaker than for the medial and lateral TF region (up to 0.32 mm and 0.35 mm per grade, respectively; Fig. 5). Lateral TF BMLs also were associated with somewhat thicker cartilage in the medial anterior femur.

Subregional analyses in the PF compartment indicated a negative association between BMLs in the lateral patella and especially the lateral anterior femur, not seen on the medial side (Supplementary Figure S3). For medial TF BMLs, the subregions generally all showed a similar pattern, indicating a negative association with cartilage thickness throughout the entire medial TF compartment (Supplementary Figure S4). Similar results were seen for the association of lateral TF BMLs with lateral cartilage thickness (Supplementary Figure S5). BMLs in the lateral anterior femur and lateral patella were associated with significantly thicker cartilage in the medial tibia.

### Osteophytes

**Fig. 6:**
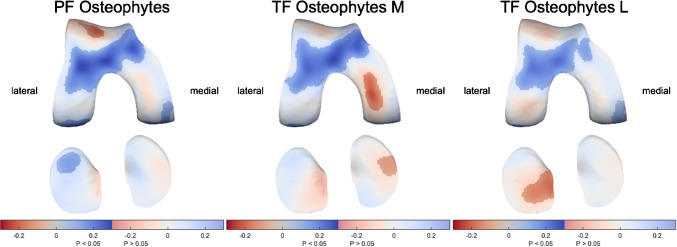
The association of osteophytes with cartilage thickness distribution. Statistically significant differences (*p* < 0.05) are indicated by the unmasked areas, while washed-out areas indicate non-significant differences (*p* > 0.05). Blue indicates an increase and red a decrease in cartilage thickness as a result of a higher patellofemoral (PF), medial (M) tibiofemoral (TF), or lateral (L) TF osteophyte grade (all up to 0.24-mm thinner local cartilage and up to 0.3-mm thicker cartilage in the anterior femur per score increase for all three regions)

While osteophytes in the PF region, medial TF region, and lateral TF region showed negative associations with cartilage thickness in the same region (up to 0.24 mm per grade), a more pronounced association with cartilage thickness in the anterior femur was seen (Fig. 6). Osteophytes in any region were associated with significantly thicker cartilage in the medial and lateral anterior femur (up to 0.3 mm per grade).

Subregional analyses showed the positive association with anterior femoral cartilage thickness was consistent for the subregions of the PF compartment (Supplementary Figure S6), medial TF compartment (Supplementary Figure S7), and lateral TF compartment (Supplementary Figure S8). As for negative associations, osteophytes in the lateral anterior femur and especially inferior patella were associated with significantly thinner cartilage in the lateral anterior femur (Supplementary Figure S6), while osteophytes in the central medial femur and especially medial tibia were associated with significantly thinner cartilage in the medial TF compartment (Supplementary Figure S7).

## Discussion

Applying CaSM to evaluate whole-joint cartilage thickness in knee OA patients of the IMI-APPROACH showed that across the study cohort, articular cartilage was thinnest in the medial tibia and exterior region of the medial femur, while it was thickest in the lateral femur and trochlea. Furthermore, cartilage thickness was shown to be dependent on almost all factors evaluated. The negative association of KL, JSN, and meniscal extrusion with tibiofemoral cartilage was spatially distributed as expected.

Male patients were shown to have thicker cartilage throughout most of the joint, as has been reported in other studies [3, 22, 23]. However, after adjusting especially for height, this effect was only statistically significant for the trochlea in prior studies, while in our study, height seemed to be the demographic factor significantly associated with thicker medial cartilage. The positive association between height and cartilage thickness is likely due to a size effect, as larger bones tend to have thicker cartilage [24]. The limitation to subregional effects for sex and height after adjustment for all demographic variables does not correspond with previous studies on this topic but may be the result of the different methods and statistical analyses applied. Perhaps surprisingly, weight was not significantly associated with cartilage thickness after adjustment for other demographics, and while the negative dependence on age was expected, it was limited to the trochlea and anterior lateral tibia with respect to statistical significance.

The fact that higher KL grades were positively associated with anterior femoral cartilage thickness was an unexpected finding that is also seen when looking at the effect of osteophytes anywhere in the joint and to a lesser degree for meniscal extrusion. Previous studies have shown a similar effect especially in posterior femoral regions, and a previous study performing similar analyses showed higher KL grades were associated with thinner trochlear cartilage, except for KL 4, which showed a slight increase in trochlear cartilage thickness [25–27]. In our study, the positive association was not limited to extreme cases, as the influence of radiographic OA (ROA) vs non-ROA looked almost identical to that of KL grade, and the associations of subregional presence or absence of osteophytes showed the same effect (Supplementary Figures S6-S8). A possible explanation could be swelling of cartilage occurring in non-weight-bearing regions, an effect that has been described previously in early or posttraumatic OA [28, 29]. Increased osteophyte formation has also been reported together with cartilage thickness increase after knee joint distraction treatment, where it was speculated that osteophytosis might be a bystander effect of cartilage repair activity [30]. In the current study, it might indicate an ongoing repair effort of the joint, which fails in weight-bearing areas. Alternatively, both increased cartilage thickness and osteophytosis are observed in early acromegaly, potentially leading to misdiagnosis as osteoarthritis, although the disease is rare [31, 32]. Whether there indeed is cartilage thickening or simply decreased thinning cannot be verified in the current study and requires longitudinal follow-up of these patients. Osteophytes also seemed to have a local negative association with cartilage thickness, which is more in line with expectations according to OA progression.

BML size showed consistent negative associations with cartilage thickness throughout the whole region, regardless of in which subregion the BML was located (Supplementary Figures S3-5). BML presence and size have been shown to be associated with thinner cartilage in several previous studies, and BMLs have been shown to predict structural OA progression, particularly cartilage loss [2, 4, 33]. As such, it could be reasoned that cartilage loss is the result of BML size, but longitudinal analyses should confirm whether or not that is the case in this cohort as well. The fact that BMLs were associated with thinner cartilage in the entire corresponding region has not yet been shown so clearly in previous studies. However, exploratory additional analyses indicated that the association with BMLs and cartilage thickness in the medial and lateral tibiofemoral regions might be influenced mostly by the central tibia, since adjustment for other BMLs (i.e., including BML size of all subregions of the patellofemoral, medial tibiofemoral, or lateral tibiofemoral region in the models as independent variables) left only the central medial and lateral tibia as significantly associated with cartilage thickness (data not shown). However, this can also be the result of the central tibia being most affected by BMLs (as seen in Supplementary Table S1) and should ideally be evaluated in a larger cohort. The positive association between medial tibial cartilage thickness and BMLs in the lateral anterior femur and the lateral patella is a surprising finding that cannot easily be explained and, thus, should be evaluated further in a larger cohort and/or a cohort affected by patellofemoral OA. However, since cartilage changes progress slowly while BMLs can show rapid changes in a short period of time, finding consistent associations might be difficult even in a larger cohort [34].

This study had several limitations. First, only cross-sectional analyses were performed. Evaluating longitudinal changes as well would allow analysis of cartilage thickness changes and their dependence on concurrent pathology changes as well as prediction of cartilage morphology changes over time. This was beyond the scope of the current study but is planned in the IMI-APPROACH cohort. Also, while all patients in the IMI-APPROACH cohort had clinical knee OA, only half (54%) had ROA. Split analyses for patients with and without ROA could not be performed, since pathologies were rarely observed in those without ROA. As such, future analysis in a larger cohort would be useful to show effects in ROA and non-ROA patients separately. Lastly, this paper evaluated a large number of variables as a first exploratory evaluation and as such did not include multiple combinations of subscores or more complex statistical models; future analyses should look into more elaborate analyses to follow up on specific findings presented here, such as the associations of BMLs and osteophytes with cartilage thickness patterns. Such evaluations could include different uses of the MOAKS grading (e.g., adding subregional scores to provide a continuous regional measure), including interaction terms in the statistical models, or using non-linear models.

In conclusion, 3D CaSM allows visualization of differences in cartilage thickness between disease states and demographics without a need for arbitrary ROI definition, potentially identifying new factors or regions that warrant further research. Male sex and height were positively associated with trochlear and patellofemoral cartilage thickness, respectively, while age was negatively associated with trochlear thickness. Tibiofemoral pathology factors show both positive trochlear and negative local tibiofemoral associations, which aligns with previous reports of the incidence of both regional thickening and thinning of articular cartilage in OA [35, 36]. The present study supports the value of the CaSM technique in terms of demonstrating expected relationships with pathology, while also highlighting opportunities for generating novel insight.

## Supplementary Information

Below is the link to the electronic supplementary material.

Supplementary file1 (DOCX 13697 KB)

## Data Availability

All relevant data are available upon request by sending an email to the Rheumatology department of the UMC Utrecht (urrci@umcutrecht.nl). This is a non-author email address that allows for maintenance of long-term data accessibility.
