# Supplementary material for: Cartilage thickness distribution and its dependence on demographic, radiographic, and MRI structural pathology in knee osteoarthritis—data from the IMI-APPROACH cohort
Source: Skeletal Radiol. 2025 Mar 21;54(10):2025–34. doi: 10.1007/s00256-025-04907-4 (PMC12361290; doi:10.1007/s00256-025-04907-4)
Supplement: Supplementary file 1 — Supplementary file1 (DOCX 13697 KB) [file 256_2025_4907_MOESM1_ESM.docx]

**Supplementary material**

Supplementary Table S1: subregional pathology overview

| **Parameter** | **Total**  **(n=287)** | **Parameter** | **Total**  **(n=287)** |
| --- | --- | --- | --- |
| *Meniscal extrusion medial* | | *Meniscal extrusion lateral* | |
| Anterior | 85 (30) | Anterior | 9 (3) * |
| Medial | 157 (57) | Lateral | 44 (16) |
| *BMLs medial* | | *BMLs lateral* | |
| Femur Central | 67 (24) | Femur Central | 27 (9) |
| Femur Posterior | 37 (13) | Femur Posterior | 33 (12) |
| Tibia Anterior | 27 (10) | Tibia Anterior | 6 (2) * |
| Tibia Central | 54 (20) | Tibia Central | 44 (15) |
| Tibia Posterior | 31 (11) | Tibia Posterior | 41 (15) |
| *Osteophytes medial* | | *Osteophytes lateral* | |
| Femur Central | 148 (52) | Femur Central | 159 (56) |
| Femur Posterior | 203 (72) | Femur Posterior | 61 (22) |
| Tibia | 123 (44) | Tibia | 126 (45) |
| *BMLs patellofemoral* | | *Osteophytes patellofemoral* | |
| Femur Lateral Anterior | 55 (19) | Femur Lateral Anterior | 79 (18) |
| Femur Medial Anterior | 56 (20) | Femur Medial Anterior | 91 (32) |
| Patella Lateral | 92 (33) | Patella Lateral | 126 (45) |
| Patella Medial | 88 (31) | Patella Medial | 104 (37) |
|  |  | Patella Superior | 75 (27) |
|  |  | Patella Inferior | 88 (31) |

*Lateral anterior meniscal extrusion and lateral anterior tibia BMLs were not present in enough patients to analyze individually in subregional analyses; however they were still taken into account in the regional analyses.

Supplementary Figure S1: The association of demographics with cartilage thickness distribution in univariable models.

Statistically significant differences (P<0.05) are indicated by the unmasked areas, while washed-out areas indicate non-significant differences (P>0.05). Blue indicates an increase and red a decrease in cartilage thickness as a result of male sex (*vs* female sex), higher age (per year increase), higher BMI (per kg/m^2^ increase), higher weight (per kg increase), higher height (per cm increase).

Note: color scale differs between models.

Supplementary Figure S2: The association of subregional medial meniscal extrusion with cartilage thickness distribution.

All models are corrected for age, sex and height; extrusion is included as a binary parameter (presence/absence).

M: medial; L: lateral.

Supplementary Figure S3: The association of subregional patellofemoral bone marrow lesions (BMLs) with cartilage thickness distribution.

All models are corrected for age, sex and height; BMLs are included as a binary parameter (presence/absence).

FMA: femur medial anterior; PM: patella medial; FLA: femur lateral anterior; PL: patella lateral.

Supplementary Figure S4: The association of subregional medial tibiofemoral bone marrow lesions (BMLs) with cartilage thickness distribution.

All models are corrected for age, sex and height; BMLs are included as a binary parameter (presence/absence).

FMC: femur medial central; FMP: femur medial posterior; TMA: tibia medial anterior; TMC: tibia medial central; TMP: tibia medial posterior.

Supplementary Figure S5: The association of subregional lateral tibiofemoral bone marrow lesions (BMLs) with cartilage thickness distribution.

All models are corrected for age, sex and height; BMLs are included as a binary parameter (presence/absence). The anterior lateral tibia could not be separately analyzed due to the small number of patients with BMLs in this subregion.

FLC: femur lateral central; FLP: femur lateral posterior; TLC: tibia lateral central; TLP: tibia lateral posterior.

Supplementary Figure S6: The association of subregional patellofemoral osteophytes with cartilage thickness distribution.

All models are corrected for age, sex and height; osteophytes are included as a binary parameter (presence/absence).

FMA: femur medial anterior; FLA: femur lateral anterior; PM: patella medial; PL: patella lateral; PS: patella superior; PI: patella inferior.

Supplementary Figure S7: The association of subregional medial osteophytes with cartilage thickness distribution.

All models are corrected for age, sex and height; osteophytes are included as a binary parameter (presence/absence).

FMC: femur medial central; FMP: femur medial posterior; TM: tibia medial.

Supplementary Figure S8: The association of subregional lateral osteophytes with cartilage thickness distribution.

All models are corrected for age, sex and height; osteophytes are included as a binary parameter (presence/absence).

FLC: femur lateral central; FLP: femur lateral posterior; TL: tibia lateral.
